# The Body Adiposity Index is not applicable to the Brazilian adult population

**DOI:** 10.3389/fnut.2022.888507

**Published:** 2022-08-25

**Authors:** José Ronaldo Ribeiro da Costa, Roberto Fernandes da Costa, Ciro Alexandre Mercês Goncalves, Michelle Vasconcelos de Oliveira Borges, Paulo Francisco De Almeida-Neto, Gilmara Gomes De Assis, Breno Guilherme De Araujo Tinoco Cabral, Paulo Moreira Silva Dantas

**Affiliations:** ^1^Department of Physical Education, Federal University of Rio Grande do Norte, Natal, Brazil; ^2^Health Sciences Center, Federal University of Rio Grande do Norte, Natal, Brazil

**Keywords:** obesity, physical evaluation, body composition, Body Adiposity Index, fat percentual, body fat percentage, fat-free mass

## Abstract

**Background:**

Obesity is a serious disease that burdens public health systems around the world. It is a risk factor for the development of several non-communicable chronic diseases that are related to the amount and distribution of body fat. Body composition assessment using simple and low-cost techniques can help in the early detection of excess fat, allowing for the prevention and treatment of both obesity and associated diseases. Thus, identifying and proposing valid anthropometric indices for this purpose can be a great ally of health programs.

**Objective:**

To verify the validity of the Body Adiposity Index (BAI) in relation to Dual Energy X-Ray Absorptiometry (DXA) for estimating body fat percentage in Brazilian adults, as well as to propose a new mathematical model to estimate the fat-free mass of this population.

**Methods:**

In a cross-sectional study, 424 subjects (of which 220 were women), aged between 20 and 59 years, were evaluated by BAI and DXA, then randomly divided into two groups stratified by sex: the development group (*n* = 283) and the cross-validation group (*n* = 141). Statistical analyses to test the validity of BAI as a predictor of fat mass, in addition to proposing a new mathematical model for estimating fat-free mass, using DXA as a reference method. The analysis included paired *t-*test, stepwise multiple regression, coefficient of concordance correlation, and Bland-Altman plots.

**Results:**

The BAI validity analysis showed a low correlation coefficient of agreement [CCC = 0.626; ρ (precision) = 0.795; C_b_(accuracy) = 0.787]; in addition, the mean difference in the Bland-Altman plot was different from zero in the cross-validation group (*p* < 0.01) and limits of agreement (LOA) ranged between−8.0 and 14.4 kg, indicating a poor agreement between the BAI and the reference method. The new mathematical model for estimating FFM showed a high correlation coefficient of agreement (CCC = 0.952; ρ = 0.953; C_b_ = 0.999), in addition to acceptable LOA in the Bland-Altman plot (-6.7 and 6.7).

**Conclusion:**

In the studied sample, the BAI showed low validity for estimating body fat, while the new proposed model was found to be a good option to assess the body composition of Brazilian adults.

## Introduction

Obesity is a severe disease overloading public health systems worldwide. It is an important risk factor for developing chronic diseases such as hypertension and diabetes, as well as cardiovascular and cerebrovascular events (1). Such a risk impact on secondary conditions is related to body fat distribution, i.e., an abnormal amount of body fat exerts an independent risk according to morbidity and represents a different impact in relation to other forms of excessive weight (2, 3).

Body composition assessments, i.e., determining the proportions of fat mass and fat-free mass, are an essential factor for prescribing and monitoring dietary and exercise programs, as well as verifying the response to such programs and health treatments (4). The most accurate method for the body composition assessment is the four-compartment model (4C) which is the criterion method for assessing fat mass (FM) and fat-free mass (FFM) at the molecular level, given that the variability of the main FFM components (water, protein, and minerals) is assessed (5). Furthermore, techniques such as magnetic resonance imaging (MRI) and dual-energy X-ray absorptiometry (DXA) rely on high-cost equipment and qualified personnel, so relatively simple and low operating cost techniques such as anthropometry and bioimpedance have been preferred in clinical and field settings (5–7).

Several mathematical models and anthropometric indices have been proposed to estimate different body components and health risk factors, in order to simplify the role of health professionals in clinical practice or field assessments (8–10).

Previously, in a study by Bergman et al. (11), a new anthropometric index for the estimation of body fat mass was presented, called the Body Adiposity Index (BAI). In this study, the BAI was successfully validated using DXA as a reference method, for the determination of body fat percentage (BF%) in a sample of American and Mexican men and women. Subsequently, Barreira et al. (12) tested the BAI in a large sample of 3,851 subjects in Baton Rouge (USA) and concluded that the BAI is not a valid predictor of BF%. Likewise, a study conducted in Brazil (13) also pointed out that the BAI is not an efficient predictor of BF% in young Brazilian athletes of both sexes.

Considering the inconsistent findings regarding the validity of the BAI for determining BF% in different population groups, we hypothesized that the BAI may not be suitable for all populations. We verified the need to test the validity of the BAI to estimate the results obtained by DXA for BF% in different Brazilian subpopulations. Thus, the objective of this study was to verify the validity of the BAI in relation to DXA for estimating BF% in Brazilian adults, as well as to propose a new mathematical model to estimate the fat-free mass of this population.

## Methods

### Sample

Four hundred and twenty-four participants (51.9% women, 20–59 years old), from the northeast region of Brazil, who were recruited through dissemination among the participants of university extension projects from the Physical Education Department of the Federal University of Rio Grande do Norte (UFRN) were included in this cross-sectional study.

After their inclusion in the study, the sample was randomly divided into two groups, i.e., the group used for the development of a mathematical model for FFM (*n* = 283) and the cross-validation group (*n* = 141). For the sample size calculation, using FFM as a primary outcome, we considered a medium to small effect size (0.10) with six predictors (independent variables), with a type I error of 5% and a power of 95%. Using these parameters, a total of 132 participants was required.

### Procedures

All data collection was conducted in a single visit by each participant to the laboratory to perform anthropometric measurements and DXA assessments, after consenting to the ethical terms approved by the Ethical Committee of the University Hospital Onofre Lopes–HUOL/UFRN–ID CODE: 34804414.7.0000.5292. Individuals with any physical deficiency, prothesis, under specific diets, or reporting diuretic problems or edema were excluded from the sample.

### Anthropometric evaluation

Weight (W) was measured using a Sanny^®^ digital scale (BL200PP, American Medical, São Bernardo do Campo, Brazil), with 0.1 kg precision. Participants were barefoot and wearing light clothes. The height (Ht) was measured using the stadiometer Caprice Sanny^®^ (American Medical do Brasil, São Bernardo do Campo, Brasil) with 0.1 cm precision, and participants were barefoot, in an orthostatic position. Hip circumference (HC) was measured using a Sanny^®^ measuring tape with 0.1 cm precision, at the level of the maximum extension of the buttocks posteriorly in a horizontal plane, as described in the original BAI article (11). Waist circumference (WC) was measured at the midpoint between the iliac crest and the lower border of the last rib, using a Sanny^®^ anthropometric metal tape measure with 0.1 cm precision, with the participants standing and the tape measure over bare skin at the measurement site. The body mass index (BMI) was determined as the body mass (kg)/height^2^ (m).

### Body adiposity index

The BAI was calculated using the height (Ht) and the hip circumference (HC) in Bergman et al. (11) equation as:


$$\begin{array}{l} BAI\;=\frac{HC\;\left(cm\right)}{Ht\;\left(m\right)\;\sqrt{Ht\;\left(m\right)}}-18 \end{array}$$

### Dual-energy X-ray absorptiometry

The DXA scan was performed using the Lunar Prodigy, model NRL 41990 (GE Lunar^®^, Madison, WI, USA) with participants in the supine position, feet attached and stabilized to the stretcher, and hands in pronation. Measurements were performed following the recommendations proposed by Nana et al. (14). The body composition was determined by enCORE software (GE Healthcare^®^, version 15.0, Madison, WI, USA).

### Statistics

The Kolmogorov-Smirnov test was applied to verify the normal distribution of the data. The descriptive analysis consisted of the mean and standard deviation of all study variables, and the comparisons between groups were performed using Student's *t*-test for independent samples.

To test the validity of the BAI to estimate BF%, the means of the results obtained by BAI and measured by DXA were compared using the paired *t*-test. In addition, Pearson's correlation coefficient (r), coefficient of determination (*r*^2^), and standard error of the estimate (SEE) were calculated. The approach proposed by Lin (15) was used for the concordance correlation coefficient (CCC) analysis to verify the validity (ρ) and accuracy (C_b_) between the estimated and measured BF% values.

The stepwise multiple regression analysis was used to propose the new mathematical model for FFM. The stepwise regression analysis was conducted using FFM obtained by DXA as a dependent variable and age, sex, weight, height, hip circumference, and waist circumference as possible independent variables. During model development, the normality of the residuals and homogeneity of variance were tested. Significance at *p* < 0.05 was established as the criterion for inclusion of a predictor, whereas removal criteria were set at *p* > 0.1. If more than one variable remained in the model, and to assess multicollinearity, a variance inflation factor (VIF) and the tolerance (reciprocal of VIF) were calculated for each independent variable, and a VIF < 10 or tolerance higher than 0.1 was considered appropriate (16, 17). To verify the validity of the proposed model, the same approach as described for the BAI was used. For the cross-validation of the new model proposed in this study, a multiple regression analysis was performed. In turn, the new model accuracy was evaluated using pure error (PE), which was calculated as the square root of the mean of the sum of squared differences between the measurement and estimate of FFM (18). The Bland-Altman (19) plots were used to verify bias and concordance between FFM measurement and estimate, in which the limits of agreement (LOA) were defined as the mean of differences ± 1.96 standard deviations, including the analysis of the correlation between the mean and the difference of the methods. Analyses were carried out with the statistical package SPSS v.20.0 (SPSS Inc., IBM Corp., Armonk, New York, NY, United States) and MedCalc version 12.5.0. Statistical significance of p < 0.05 was considered for all tests.

## Results

Table 1 describes the physical characteristics and body composition variables for the developmental and cross-validation groups, as well as for the whole sample with no differences observed between the two groups (i.e., developmental and cross-validation) (*p* > 0.05).

**Table 1:** Descriptive characteristics and body composition of development and cross-validation groups (mean ± sd).

	**Development group (DG)**			**Cross-validation group (CVG)**		
	**Male**	**Female**	**Whole sample**	**Male**	**Female**	**Whole sample**
	**(*n* = 136)**	**(*n* = 147)**	**(*n* = 283)**	**(*n* = 68)**	**(*n* = 73)**	**(*n* = 141)**
Age (yrs)	36.6, 12.5	38.7, 13.0	37.7, 12.8	38.8, 12.8	40.0, 13.0	39.4, 12.9
Weight (kg)	78.4, 14.1	65.7, 13.3	71.8, 15.1	80.4, 13.0	66.8, 10.9	73.4, 13.7
Height (cm)	174.9, 7.4	161.3, 6.3	167.9, 9.6	174.5, 7, 2	161.6, 6.9	167.8, 9.6
BMI (kg/m^2^)	25.6, 4.0	25.2, 4.9	25.4, 4.4	26.4, 3.9	25.7, 4.4	26.0, 4.2
FM (kg)	19.2, 8.6	25.1, 9.2	22.2, 9.4	21.0, 8.0	25.8, 8.6	23.5, 8.6
FM (%)	23.8, 7.2	37.2, 7.0	30.7, 9.8	25.5, 7.0	37.9, 7.7	31.9, 9.6
FFM (kg)	59.2, 9.1	40.6, 5.9	49.6, 12.0	59.4, 8.1	41.0, 5.9	49.9, 11.6
Hip circumference (cm)	99.6, 7.6	100.4, 9.5	100.0, 8.6	99.3, 8.6	102.4, 8.6	100.9, 8.7
Waist circumference (cm)	88.2, 11.7	82.3, 12.9	85.1, 12.6	90.6, 10.8	83.6, 14.6	87.0, 13.3
BAI (BF%)	25.1, 3.4	31.1, 5.0	28.2, 5.2	25.2, 4.2	32.0, 5.3	28.2, 5.2

BMI, Body Mass Index; FM, fat mass; FFM, fat-free mass; BAI, Body Adiposity Index; BF%, body fat percentage.

When testing the validity of the BAI to estimate the body fat percentage, although the correlation with DXA was high (r = 0.795; *p* < 0.01), it was found that there was a significant difference when the mean results were compared with the measurements obtained by DXA (DXA = 30.7 + 9.8; BAI = 28.2 + 5.2; *p* < 0.01). The other validity criteria used are presented in Table 2, together with the performance of the cross-validation of the model proposed in this study for the estimation of fat-free mass.

**Table 2:** Cross-validation of FFM predictive new model, and validation of BAI for BF%.

			**CCC Analysis**				
	**FFM (kg)**	*p*-value*	**CCC**	**ρ**	**C_b_**	**r^2^**	**PE (kg)**
DXA	49.9 ± 11.6						
New model	49.5 ± 11.5	0.844	0.952	0.953	0.999	0.91	3.40
			**CCC Analysis**				
	**BF%**	***p***-value*	**CCC**	ρ	**C**_b_	**r** ^2^	**PE (%)**
DXA	30.7 ± 9.8						
BAI	28.2 ± 5.2	<0,001	0.625	0.795	0.787	0.63	6.54

BAI, Body Adiposity Index; FFM, fat-free mass; BF%, body fat percentage; CCC, Concordance Correlation Coefficient; ρ, accuracy; C_b_, validity; PE, pure error. ^*^Differences between predictive models and reference method by paired t-test.

Table 3 shows the regression model for predicting FFM (kg). The possible independent variables used were: age (years), sex, weight (kg), height (cm), hip circumference (cm), and waist circumference (cm). Only the variables that contributed to the estimates using a backward stepwise approach were used in the model. The performance of the developed model can be observed by the high coefficient of determination (r^2^ = 0.91) and low standard error of the estimate (SEE = 3.67 kg).

**Table 3:** Regression model for the prediction of FFM (kg).

**Variables included in the model**	**Regression coefficient**	**r^2^**	**SEE**	***p*-value**	**Collinearity statistics**	
					**Tolerance**	**VIF**
Constant	+26,771			<0.001		
Height	+0.143	0.648^a^	7.138	<0.001	0.293	3.409
Weight	+0.725	0.806^b^	5.307	<0.001	0.130	7.695
Sex	−7.942	0.884^c^	4.118	<0.001	0.355	2.816
Age	−0.087	0.893^d^	3.963	<0.001	0.799	1.252
Hip Circumference	−0.328	0.902^e^	3.791	<0.001	0.182	5.489
Waist Circumference	−0.154	0.909^f^	3.669	<0.001	0.251	3.989

SEE, standard error of the estimate; VIF, variance inflation factor. Predictors: ^a^(Constant), Height; ^b^(Constant), Height, Weight; ^c^(Constant), Height, Weight, Sex; ^d^(Constant), Height, Weight, Sex, Age; ^e^(Constant), Height, Weight, Sex, Age, Hip Circumference; ^f^(Constant), Height, Weight, Sex, Age, Hip Circumference, Waist Circumference. The r^2^ change was significant for a, b, c, d, e, and f.

The resulting prediction model included is shown below, including FFM (fat-free mass) in kg, height (Ht) in cm, weight (W) in kg, sex (male = 0; female = 1), age in years, hip circumference (HC) in centimeters and waist circumference (WC) in cm:


$$\begin{array}{l} \text{FFM = }26.771\;+\;0.143Ht\;+\;0.725\;W\;-\;7.942Sex\;-\;0.087Age\\ \;-0.328\text{HC }-\;0.154\text{ WC} \end{array}$$

From the results of FFM, it is possible to calculate FM in kilograms by subtracting FFM from body mass (FM = BM – FFM). Then, it is also possible to calculate body fat percentage by the mathematical expression: BF% = (FM × 100)/BM.

Estimated FFM by the new model developed in this study did not present significant differences in comparison with the value determined by DXA for both the development and cross-validation groups. All parameters used for proposing and validating the model confirmed their validity. Additionally, no association was found between the mean and the difference in the methods (r = 0.08; *p* = 0.356).

**Figure 1:**
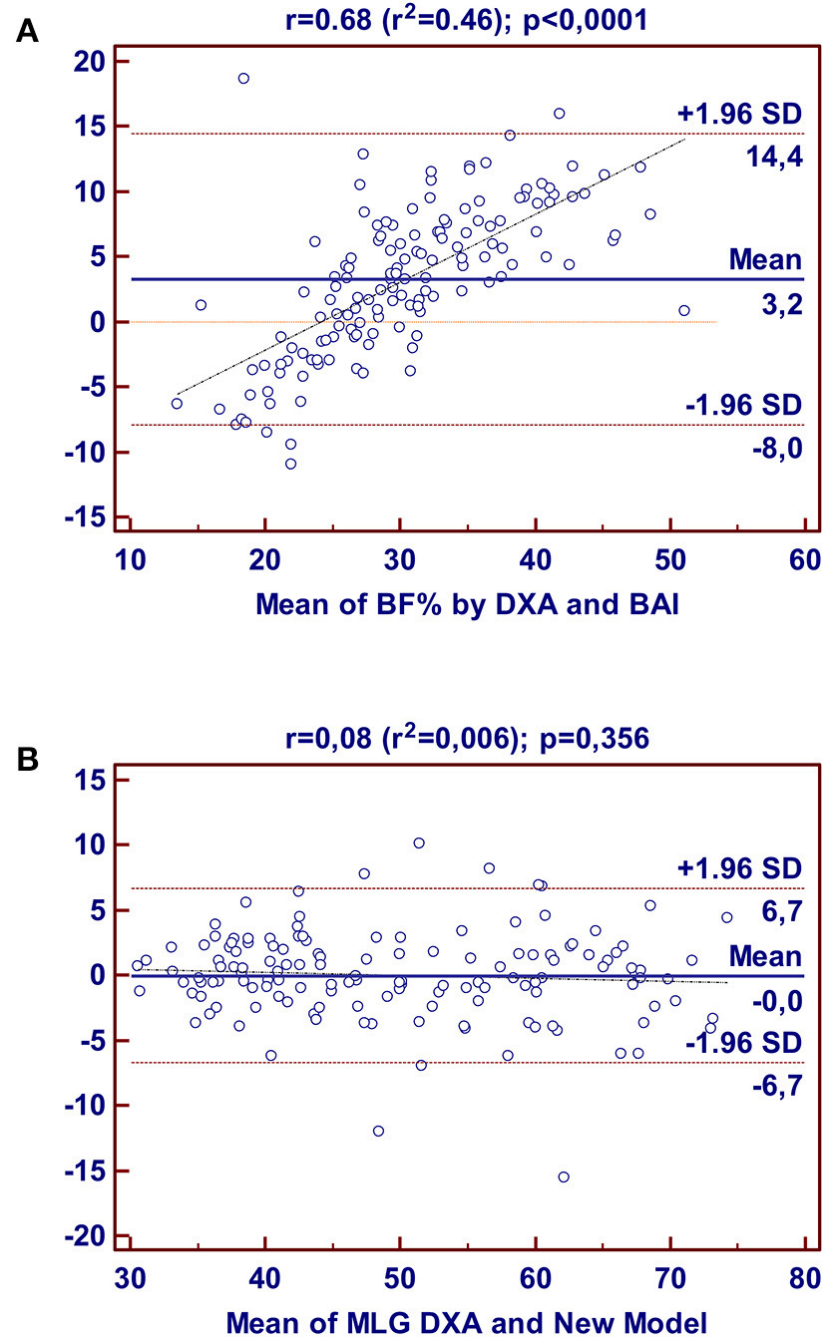
Bland-Altman plots for the concordance limits between values determined by the reference method (DXA) and BAI for BF% **(A)**; and between values determined by the reference method (DXA) and New Model for FFM **(B)** in Brazilian adults, derived in this study.

Figure 1 presents the LOA in the cross-validation group for BF% between the standard method (DXA) and the BAI, and the LOA for FFM between DXA and the new model developed in this study.

The mean difference in the Bland-Altman plot was different from zero in the cross-validation group for BAI (*p* = 0.006) but was not different from zero for FFM by the new model (*p* = 0.728). For BAI, the LOA ranged between−8.0 and 14.4 for the body fat percentage, while the LOA ranged between−6.7 and 6.7 kg for fat-free mass, indicating poor agreement between BAI and DXA, but an acceptable agreement between the new model and the reference method.

## Discussion

The objective of the present study was to evaluate the validity of the BAI, using DXA as a reference, to estimate body fat percentage in subjects from the Brazilian population. The results did not confirm the validity of the BAI for estimating body fat in Brazilian adults, which justifies the need to create new mathematical models to estimate the body composition of this population. Thus, we developed and cross-validated a model for estimating fat-free mass through anthropometric measurements, using DXA as a reference method.

The BAI is an alternative DXA-based low-cost method for determining body adiposity with a simplified protocol and acceptable accuracy verified in different populations. However, except for the applicability of BAI for the diagnosis of excessive body fat, there is no consensus in the literature regarding its validity in different populations (7, 20).

The equations for the development of BAI are based on body height and hip circumference since these variables correlate with the BF% verified by DXA. The original study–named BetaGene–used men and women from the adult Mexican-American population of the USA. Different populations show distinct anthropometric characteristics, including body fat distribution (21, 22). Thus, an index based on a certain population may not attend to the specific characteristics of another population. Such conflict in the applicability of BAI has been evidenced by several studies (13, 20, 23–25). These reports consistently show that the BAI does not offer a valid estimation of BF% in subpopulations such as Caucasians, Europeans, adult Americans, female athletes, Brazilian women, and children. In line with the above, the main finding in our study was the poor validity of BAI, in relation to DXA, to estimate BF% in Brazilian adults.

An issue that deserves to be highlighted is that the use of BAI in men and women may have different associations with the body fat values obtained by DXA, which may be related to the distribution of body fat, as it is more concentrated in the gluteal-femoral region in women, in contrast to the abdominal region in men (26), thus interfering with the results of the BAI, which has hip circumference as one of its variables. In this way, perhaps a model that also uses waist circumference would provide better results.

Several studies have shown unsatisfactory results with the use of the BAI. Miazgowski et al. (27) analyzed 234 Caucasian women aged between 20 and 40 years and found a moderate correlation between the BF% values provided by the BAI and DXA. The validity of the BAI was also tested in 106 Asian adults by Lam et al. (24), and their results showed that the values obtained by BAI underestimated the BF% of individuals by an average of 5.77% in relation to DXA. Furthermore, Freedman et al. (28) found that BAI values reported a 75% overestimation in BF% of men and a 70% underestimation in women in a large sample of 1,151 adults of different ethnicities (37% Caucasian, 27% Black, 25% Hispanic, 8% Asian, and 3% others).

Differences in fat distribution in different ethnicities have been previously reported as being associated with environmental and cultural factors, such as nutrition habits and physical activity (21, 22). In this sense, despite the original study's validation of the BAI for the evaluation of BF% in North Americans, it seems that this validation was biased by the ethnicity of the sample, which was mostly Black.

The present study also found that there is a large difference in the limits of agreement between the BAI and DXA, according to the Bland-Altman plot. It was verified that the BAI might underestimate BF% as much as 8.0% or overestimate it up to 14.4% in adult Brazilians, which indicates that the BAI is not an adequate tool for the determination of BF% in healthy adult Brazilians. Convergently, Cerqueira et al. (20) previously detected a tendency for an overestimation when applying the BAI for the determination of BF% in Brazilians with low fat mass, as well as an underestimation in Brazilians with obesity.

Another study carried out in Brazil analyzed the validity of the BAI to estimate the BF% of 144 adults with severe obesity (candidates for bariatric surgery), using air displacement plethysmography (BOD POD^®^) as the standard technique (29). The authors showed poor validity of the BAI and LOA very close to those of the present study for the percentage of body fat (-7.48 to 14.84). Thus, the authors chose to develop a new mathematical model for the study population.

In this sense, the creation of a mathematical model for estimating body composition in the population of the present study, using simple anthropometric measurements, seems to be a viable way to obtain better results than the BAI. Thus, we developed and tested the validity of several models, and the one that presented the best results of validity and cross-validation was the one that proposed the estimation of fat-free mass and included as dependent variables height, weight, sex, age, hip circumference, and waist circumference, with excellent prediction performance observed by the high coefficient of determination (r^2^ = 0.91) and low standard error of the estimate (SEE = 3.67 kg).

The low cost and ease of use may explain the number of studies that have been carried out to propose anthropometric mathematical models for estimating body composition in different groups or to validate existing ones (30–35). However, most of these equations were proposed to meet the specificities of the group under study, such as breast-feeding children (30), children (34), adolescent athletes (33), healthy adults (31), or those with chronic diseases (32) and elite athletes (35), among many others, which cannot be generalized to groups other than the population of origin.

Considering that there is no consensus on generalizable anthropometric prediction equations to validly estimate body composition, a comprehensive study was carried out to develop and validate practical anthropometric predictions for lean body mass, fat mass, and percent fat in adults (men, *n* = 7,531; women, *n* = 6,534) participating in the National Health and Nutrition Examination Survey 1999-2006 (36). The authors derived several regression models, with different anthropometric measurements, including circumferences and skinfolds, and concluded that a practical equation including age, race, height, weight, and waist circumference had a high predictive ability for lean body mass and fat mass and that the inclusion of other circumferences and skinfold thickness slightly improved the prediction model.

There have been few studies carried out in Brazil with the aim of proposing mathematical models based on circumference measurements to estimate body composition. In the same geographic region of the country where we carried out our study, another study was previously carried out that proposed predictive equations for fat mass and fat-free mass of adolescents aged 10 to 16 years, based on anthropometric measurements, and concluded that the equations developed to estimate fat mass in females and fat-free body mass in all genders had high adjusted coefficients of determination (37).

More recently, in southern Brazil, a study was carried out to propose mathematical models for estimating the percentage of body fat in women aged 18 to 35 years, based on body circumferences (38). However, the authors only used Pearson's correlation coefficient and the paired *t*-test, in relation to DXA as a reference method, and lacked more robust statistical analyses to validate and carry out the cross-validation of the developed models.

In the same year, a mathematical model was developed to estimate the BF% of Brazilian subjects with severe obesity, and candidates for bariatric surgery, demonstrating high validity and limits of agreement similar to the present study, but this model does not apply to our sample since the anthropometric characteristics of the source population are very different from our sample (29).

This study has several strengths. To our knowledge, this is the first study to develop and cross-validate a predictive equation for FFM by body circumference, using DXA as a reference method, in Brazilian adults in the northeast region. The mathematical model developed in our study showed a high coefficient of determination and good limits of agreement in relation to the reference method, and all the parameters used for the proposition and cross-validation of the model confirmed its validity for the population studied (15, 18, 19, 39), which can be used to monitor changes in FFM resulting from dietary and exercise programs (40).

However, there are limitations to our study that must be addressed. The sample included adults from only one region of the country, and ethnicity was not assessed. Other studies carried out in Brazil for the development of predictive equations by anthropometry (38, 41) also used ethnically mixed samples, miscegenation, and ethnic differences, which suggests the need to validate the equation proposed in the study in other regions of the country and with subjects from different ethnic origins. Another important issue concerns the standard technique used. The 4C model is the most appropriate reference method to assess FM and FFM at the molecular level (5). However, due to the complexity of the technique (42), the use of DXA to derive anthropometric equations has been widely accepted (7, 34, 36, 37). It is noteworthy that the new equations are only useful for Brazilian adults with similar characteristics. In addition, more research should be carried out to test the accuracy of the new model in tracking FFM.

## Conclusion

In the studied sample, the BAI showed low validity for estimating body fat, while the new proposed model proved to be a good option to assess the body composition of Brazilian adults. However, we are aware that the validity of the proposed model must be tested in other regions of the country and in other population groups in order to verify its applicability.

## Data availability statement

The datasets presented in this study can be found in online repositories. The names of the repository/repositories and accession number(s) can be found in the article/supplementary material.

## Ethics statement

The studies involving human participants were reviewed and approved by Ethical Committee of the University Hospital Onofre Lopes–HUOL/UFRN ID CODE: 34804414.7.0000.5292. The patients/participants provided their written informed consent to participate in this study.

## Author contributions

JR: responsible for the concept/design, the data collection, the data analysis/interpretation, and drafting the article. RC: responsible for the interpretation and drafting the article. CG and MO: responsible for data collection, drafting the article, and critical revision of the article. PA-N and GD: responsible for translate to English, drafting the article, and critical revision of the article. BC: responsible for project supervision, data analysis/interpretation, and drafting the article. PD: responsible for the concept/design, project supervision, the data collection, drafting the article, and critical revision of the article.

## Conflict of interest

The authors declare that the research was conducted in the absence of any commercial or financial relationships that could be construed as a potential conflict of interest.

## Publisher's note

All claims expressed in this article are solely those of the authors and do not necessarily represent those of their affiliated organizations, or those of the publisher, the editors and the reviewers. Any product that may be evaluated in this article, or claim that may be made by its manufacturer, is not guaranteed or endorsed by the publisher.

## References

[1] Bastien M Poirier P Lemieux I Després J-P. Overview of epidemiology and contribution of obesity to cardiovascular disease. Prog Cardiovasc Dis. (2014) 56:369–81. 10.1016/j.pcad.2013.10.016 24438728

[2] Cristóvão MF Sato APS Fujimori E. Excesso de peso e obesidade abdominal em mulheres atendidas em Unidade da Estratégia Saúde da Família. Revista da Escola de Enfermagem da USP. (2011) 45:1667–72. 10.1590/S0080-62342011000800005 22569652

[3] Pinho CPS Diniz AdS Arruda IKGd Batista Filho M Coelho PC Sequeira LAdS. Prevalencia y factores asociados a la obesidad abdominal en individuos en una franja de edad de 25 a 59 años del estado de Pernambuco, Brasil. Cad Saúde Pública. (2013) 29:313–24. 10.1590/S0102-311X2013000200018 23459817

[4] da Costa RF Masset KVdSB de Sousa EC Cabral BGdAT Dantas PMS. Development and cross-validation of predictive equations of fat-free mass by bioelectrical impedance for Brazilian men aged 20 to 59 years old/Desenvolvimento e validacao cruzada de equacoes preditivas de massa livre de gordura por bioimpedanciometria, para homens brasileiros de 20 a 59 anos de idade. Motricidade. (2018) 14:26–33. 10.6063/motricidade.16232

[5] Heymsfield SB Ebbeling CB Zheng J Pietrobelli A Strauss BJ Silva AM. Multi-component molecular-level body composition reference methods: evolving concepts and future directions. Obes Rev. (2015) 16:282–94. 10.1111/obr.12261 25645009 PMC4464774

[6] Kuriyan R. Body composition techniques. Indian J Med Res. (2018) 148:648–58. 10.4103/ijmr.IJMR_1777_18 30666990 PMC6366261

[7] de Macêdo Cesário T de Almeida-Neto PF de Matos DG Wells J Aidar FJ Costa RF. Body adiposity index to analyze the percentage of fat in young men aged between 7 and 17 years. Am J Hum Biol. (2021) 34:e23599. 10.1002/ajhb.23599 33763955

[8] Gómez-Ambrosi J Silva C Catalán V Rodríguez A Galofré JC Escalada J. Clinical usefulness of a new equation for estimating body fat. Diabetes Care. (2012) 35:383–8. 10.2337/dc11-1334 22179957 PMC3263863

[9] Thomas DM Bredlau C Bosy-Westphal A Mueller M Shen W Gallagher D. Relationships between body roundness with body fat and visceral adipose tissue emerging from a new geometrical model. Obesity. (2013) 21:2264–71. 10.1002/oby.20408 23519954 PMC3692604

[10] Woolcott OO Bergman RN. Relative fat mass (RFM) as a new estimator of whole-body fat percentage– a cross-sectional study in American adult individuals. Sci Rep. (2018) 8:1–11. 10.1038/s41598-018-29362-1 30030479 PMC6054651

[11] Bergman RN Stefanovski D Buchanan TA Sumner AE Reynolds JC Sebring NG. A better index of body adiposity. Obesity. (2011) 19:1083–9. 10.1038/oby.2011.38 21372804 PMC3275633

[12] Barreira TV Harrington DM Staiano AE Heymsfield SB Katzmarzyk PT. Body adiposity index, body mass index, and body fat in white and black adults. JAMA. (2011) 306:828–30. 10.1001/jama.2011.1189 21862743 PMC3951848

[13] de Macêdo Cesário T de Almeida-Neto PF de Matos DG Wells J Aidar FJ de Araujo Tinoco Cabral BG. Evaluation of the body adiposity index against dual-energy X-ray absorptiometry for assessing body composition in children and adolescents. Am J Hum Biol. (2021) 33:e23503. 10.1002/ajhb.23503 32918370

[14] Nana A Slater GJ Stewart AD Burke LM. Methodology review: using dual-energy X-ray absorptiometry (DXA) for the assessment of body composition in athletes and active people. Int J Sport Nutr Exerc Metab. (2015) 25:198–215. 10.1123/ijsnem.2013-0228 25029265

[15] Lin LI. A concordance correlation coefficient to evaluate reproducibility. Biometrics. (1989) 45:255–68. 2720055

[16] Guo S Chumlea WC. Statistical methods for the development and testing of predictive equations. Human Body Composit. (1996) 10:191–202.

[17] Ndagire CT Muyonga JH Odur B Nakimbugwe D. Prediction equations for body composition of children and adolescents aged 8-19 years in Uganda using deuterium dilution as the reference technique. Clin Nutr ESPEN. (2018) 28:103–9. 10.1016/j.clnesp.2018.09.004 30390864

[18] Sun SS Chumlea WC Heymsfield SB Lukaski HC Schoeller D Friedl K. Development of bioelectrical impedance analysis prediction equations for body composition with the use of a multicomponent model for use in epidemiologic surveys. Am J Clin Nutr. (2003) 77:331–40. 10.1093/ajcn/77.2.331 12540391

[19] Bland JM Altman D. Statistical methods for assessing agreement between two methods of clinical measurement. Lancet. (1986) 327:307–10. 10.1016/S0140-6736(86)90837-8 2868172

[20] Cerqueira M Amorim P Magalhaes F Castro E Franco F Franceschini S. Validity of body adiposity index in predicting body fat in a sample of Brazilian women. Obesity. (2013) 21:E696–E9. 10.1002/oby.20543 23804594

[21] Heymsfield SB Peterson CM Thomas DM Heo M Schuna Jr J. Why are there race/ethnic differences in adult body mass index–adiposity relationships? a quantitative critical review. Obes Rev. (2016) 17:262–75. 10.1111/obr.12358 26663309 PMC4968570

[22] Jensen B Moritoyo T Kaufer-Horwitz M Peine S Norman K Maisch MJ. Ethnic differences in fat and muscle mass and their implication for interpretation of bioelectrical impedance vector analysis. Appl Physiol Nutr Metab. (2019) 44:619–26. 10.1139/apnm-2018-0276 30354265

[23] Johnson W Chumlea WC Czerwinski SA Demerath EW. Concordance of the recently published body adiposity index with measured body fat percent in European-American adults. Obesity. (2012) 20:900–3. 10.1038/oby.2011.346 22095112 PMC3988697

[24] Lam BCC Lim SC Wong MTK Shum E Ho CY Bosco JIE. A method comparison study to validate a novel parameter of obesity, the body adiposity index, in Chinese subjects. Obesity. (2013) 21:E634–E9. 10.1002/oby.20504 23630126

[25] Vinknes KJ Elshorbagy AK Drevon CA Gjesdal CG Tell GS Nygård O. Evaluation of the body adiposity index in a Caucasian population: the hordaland health study. Am J Epidemiol. (2013) 177:586–92. 10.1093/aje/kws271 23444101

[26] Chang H Simonsick EM Ferrucci L Cooper JA. Validation study of the body adiposity index as a predictor of percent body fat in older individuals: Findings from the BLSA. J Gerontol A Biol Sci Med Sci. (2014) 69:1069–75. 10.1093/gerona/glt165 24158764 PMC4158412

[27] Miazgowski T Safranow K Major-Gołuch A Krzyzanowska-Swiniarska B. Validation of a new index of body adiposity (BAI) to assess body fat in normal weight premenopausal Caucasian women. e-SPEN J. (2012) 7:e115–e8. 10.1016/j.clnme.2012.02.006

[28] Freedman DS Thornton JC Pi-Sunyer FX Heymsfield SB Wang J Pierson Jr RN. The body adiposity index (hip circumference÷ height1. 5) is not a more accurate measure of adiposity than is BMI, waist circumference, or hip circumference. Obesity. (2012) 20:2438–44. 10.1038/oby.2012.81 22484365 PMC3477292

[29] Belarmino G Torrinhas RS Sala P Horie LM Damiani L Lopes NC. A new anthropometric index for body fat estimation in patients with severe obesity. BMC Obes. (2018) 5:25. 10.1186/s40608-018-0202-8 30288293 PMC6166270

[30] Bandara T Hettiarachchi M Liyanage C Amarasena S Wong WW. Body composition among Sri Lankan infants by ^18^O dilution method and the validity of anthropometric equations to predict body fat against ^18^O dilution. BMC Pediatr. (2015) 15:52. 10.1186/s12887-015-0371-2 25943377 PMC4428108

[31] Cicone ZS Nickerson BS Choi Y-J Holmes CJ Hornikel B Fedewa MV. Generalized equations for predicting percent body fat from anthropometric measures using a criterion five-compartment model. Med Sci Sports Exerc. (2021) 53:2675–82. 10.1249/MSS.0000000000002754 34310492 PMC8785250

[32] Diniz KGD Vieira DA Colosimo EA Coelho MPP Bering T Teixeira R. Derivation and validation of a simple anthropometric equation to predict fat-free mass in patients with chronic hepatitis C. Clin Nutr. (2021) 40:1281–8. 10.1016/j.clnu.2020.08.011 32861484

[33] Gomez-Campos R Santi-Maria T Arruda M Maldonado T Albernaz A Schiavo M. Fat-free mass and bone mineral density of young soccer players: proposal of equations based on anthropometric variables. Front Psychol. (2019) 10:522. 10.3389/fpsyg.2019.00522 30984051 PMC6449479

[34] Salazar G Leyton B Aguirre C Anziani A Weisstaub G Corvalán C. Anthropometric and bioimpedance equations for fat and fat-free mass in Chilean children 7-9 years of age. Br J Nutr. (2021) 126:37–42. 10.1017/S0007114520003906 33028443

[35] Sesbreno E Slater G Mountjoy M Galloway SDR. Development of an anthropometric prediction model for fat-free mass and muscle mass in elite athletes. Int J Sport Nutr Exerc Metab. (2020) 30:174–81. 10.1123/ijsnem.2019-0232 32045882

[36] Lee DH Keum N Hu FB Orav EJ Rimm EB Sun Q. Development and validation of anthropometric prediction equations for lean body mass, fat mass and percent fat in adults using the National Health and Nutrition Examination Survey (NHANES) 1999–2006. Br J Nutr. (2017) 118:858–66. 10.1017/S0007114517002665 29110742

[37] Lyra CO Lima SCVC Lima KC Arrais RF Pedrosa LFC. Prediction equations for fat and fat-free body mass in adolescents, based on body circumferences. Ann Hum Biol. (2012) 39:275–80. 10.3109/03014460.2012.685106 22594692

[38] Salamunes ACC Stadnik AMW Neves EB. Estimation of female body fat percentage based on body circumferences. Revista Brasileira de Medicina do Esporte. (2018) 24:97–101. 10.1590/1517-86922018240218117529553036

[39] Lohman T Milliken LA Medicine ACoS. ACSM's Body Composition Assessment: Human Kinetics (2019).

[40] Costa RF Silva AM Cabral BGdAT Dantas PMS. Development and cross-validation of predictive equations for fat-free mass and lean soft tissue mass by bioelectrical impedance in Brazilian women. Eur J Clin Nutr. (2022) 76:288–96. 10.1038/s41430-021-00946-x 34230624

[41] Hoffman DJ Toro-Ramos T Sawaya AL Roberts SB Rondo P. Estimating total body fat using a skinfold prediction equation in Brazilian children. Ann Hum Biol. (2012) 39:156–60. 10.3109/03014460.2012.660989 22324842

[42] Silva A Minderico C Teixeira P Pietrobelli A Sardinha L. Body fat measurement in adolescent athletes: multicompartment molecular model comparison. Eur J Clin Nutr. (2006) 60:955–64. 10.1038/sj.ejcn.1602405 16523205

